# Laparoscopic vs. Open Abdominal Radical Hysterectomy for Cervical Cancer: A Single-Institution, Propensity Score Matching Study in China

**DOI:** 10.3389/fonc.2019.01107

**Published:** 2019-10-30

**Authors:** Zhen Yuan, Dongyan Cao, Jie Yang, Mei Yu, Keng Shen, Jiaxin Yang, Ying Zhang, Huimei Zhou

**Affiliations:** Department of Obstetrics and Gynecology, Peking Union Medical College Hospital, Peking Union Medical College, Chinese Academy of Medical Sciences, Beijing, China

**Keywords:** cervical cancer, laparoscopy, oncologic outcomes, open abdominal surgery, surgical outcomes

## Abstract

**Study Objective:** To compare the surgical and oncologic outcomes between open abdomen radical hysterectomy (ARH) and laparoscopic radical hysterectomy (LRH) for cervical cancer.

**Methods:** Retrospective observational study with propensity score matching was used to ensure balanced groups for ARH and LRH. One-hundred-and-ninety-eight women with cervical cancer, 99 treated using ARH and 99 using LRH, between January 2012 and December 2014. Outcomes included disease-free survival (DFS), overall survival (OS), intra-operative factors, post-operator recovery, urinary retention, and adverse events. Moreover, the inverse probability of the treatment weighting (IPTW) method was also used.

**Main Results:** Compared with ARH, LRH was associated with a lower volume of blood loss (*P* < 0.001) and transfusion rate (*P* < 0.001), with a broader resection of the parametrium (*P* < 0.001). Post-operatively, the time to first flatus was shorter for LRH than ARH (*P* < 0.001) but the rate of urinary retention was higher for LRH (22.2%) than ARH (8.1%; *P* = 0.009). DFS and OS were similar between groups. By IPTW, laparoscopy was also not associated with poorer survival in terms of DFS (HR 1.52, CI 0.799–2.891, *P* = 0.202) or OS (HR 0.942, HR 0.425–2.09, *P* = 0.883).

**Conclusion:** Compared with ARH, LRH provided better intra-operative and post-operative outcomes, with no significant difference in oncologic outcomes and survival. Urinary retention remains a clinical issue to improve with LRH. The technology of LRH has been improved in China to address the inconsistent results of oncologic outcomes in previous studies. Whether these improvements could be effective needs to be investigated in the future.

## Introduction

Recently, the unexpected result of a phase III prospective trial has brought a great debate within the academic arena (1). In this trial, minimally invasive radical hysterectomy was associated with a lower rate of disease-free survival (DFS) at 3-years post-surgery [91.2 vs. 97.1%, respectively; hazard ratio (HR), 3.74, with a 95% confidence interval (CI) of 1.63–8.58], and a decrease in overall survival (OS) at 3-years post-surgery (93.8 vs. 99.0%, respectively; HR 6.00 and 95% CI, 1.77–20.30). Therefore, this trial provided evidence of poorer outcomes for minimally invasive radical hysterectomy than ARH, among women with early-stage cervical cancer.

This finding is not consistent with previously reported findings. In fact, previous studies reported on the therapeutic equivalency between MIRH and ARH, with MIRH providing additional benefits of a shorter duration in hospital stay and more rapid patient recovery (2–4).

We do recognize that surgical skills and techniques vary between surgeons, and particularly between hospitals, therefore, our aim was to compare the rate of surgery- associated complications and survival between laparoscopic radical hysterectomy (LRH), which is a commonly used minimally invasive approach, and ARH at our institution.

## Materials and Methods

This study was approved by the Peking Union Medical College Hospital Ethics Review Board. Preoperatively, all patients provided written informed consent for data collection for research purposes. The data set was kept anonymous in order to protect patient privacy.

Inclusion criteria were as follows: surgery performed between January 2012 and December 2014; radical hysterectomy with lymphadenectomy; histological confirmation of squamous carcinoma, adenocarcinoma, or adenosquamous carcinoma; and regular follow-up. Patients with rare histological types of cancer and those lost to follow-up were excluded.

A radical hysterectomy includes the removal of the uterus as far as possible from the uterosacral ligaments, resection of the parameter as near as to the pelvic wall as possible, ligation of uterine vessels at the origin, and removal of 1/3 of the upper vagina (5).

Two experienced gynecological oncologists determined the clinical stage of each case, according to the International Federation of Gynecology and Obstetrics (FIGO) 2009 guidelines. Histologic diagnosis was confirmed by at least two pathologists. The following information was collected from the medical records for analysis: age, body mass index (BMI), squamous cell carcinoma antigen (SCCAg) before the initial treatment, histological type, clinical stage, surgery-related complications, treatment modality, adjuvant therapy, and outcomes. According to Common Terminology Criteria for Adverse Events (v4.0 CTCAE), the adverse events with grade ≥3 were recorded in our analysis. In this study, when the urinary catheter was removed postoperatively for the first time, a residual urine volume ≥ 100 ml was defined as urinary retention.

### Statistical Analysis

Propensity score matching was used to select patients, improving the quality of the results reported. Propensity-matched comparisons attempt to estimate the effect of a treatment, by accounting for possible factors that predict receiving the treatment, thus reducing possible selection biases. The following matching factors were used in our study: age, tumor size, stage, histologic type, lymph node metastasis, parametrium invasion, and surgical margin status. Patients undergoing LRH were matched 1:1 to patients selected to a cohort of women undergoing ARH, using a caliper width of ≤0.02 standard deviations of the logit odds of the estimated propensity score. Moreover, the inverse probability of the treatment weighting (IPTW) method was also used (6), to capture information from patients who otherwise would be discarded by 1:1 matching.

Categorical variables are summarized in frequency tables, whereas continuous variables are presented as a mean ± standard deviation or median (25th percentile−75th percentile), as appropriate for the data distribution. Frequency distributions were compared using the chi-squared tests by Fisher' s exact test, as appropriate, with mean values compared using a *t* test and median values using a non-parametric test.

DFS was calculated from the date of surgery to the date of first recurrence or last follow-up in patients and overall survival (OS) as the date of death or last contact. All of the follow-up information was censored following March 1, 2019. Survival curves were calculated using the Kaplan–Meier method and compared using the log-rank test. The data were analyzed using SPSS (version 23, IBM, Armonk, NY), Prism 7 (GraphPad Software, San Diego, CA) and RStudio (Version 1.1.463). A *p* value < 0.05 was considered statistically significant, using the two-tailed hypothesis.

## Results

**Figure 1:**
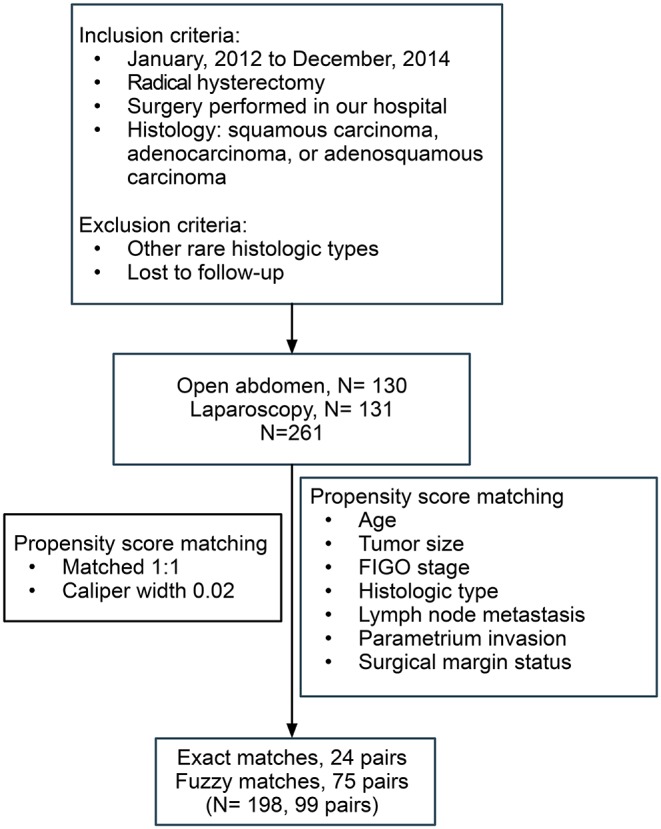
The flow of patients included in this study.

The flowchart of patient selection is shown in Figure 1. After screening and matching, 99 patients were included in each of the ARH and LRH groups. The baseline clinical characteristics of the patients forming our study group are presented in Table 1.

**Table 1:** Baseline clinical characteristics of the patients. Data are presented as number (%), mean (±SD) or median (25th percentiles- 75th percentiles).

	**Open abdomen** **(*N* = 99)**	**Laparoscopy** **(*n* = 99)**	***P* value**
Age (years)	44.56 ± 7.60	43.58 ± 8.86	0.405
BMI (kg/m^2^)	24.56 ± 1.50	24.36 ± 2.41	0.479
Previous abdominal surgery, *N* (%)	37 (37.4%)	40 (40.4%)	
SCCAg (ng/mL)	1.20 (0.70–2.90)	1.20 (0.60–2.70)	0.549
CA125 (U/ml)	22.10 (10.10–27.83)	16.50 (12.65–19.75)	0.586
**PATHOLOGICAL TYPE**			
Squamous cell cancer, *N* (%)	82 (82.8%)	82 (82.8%)	1.000
Adenocarcinoma, *N* (%)	13 (13.1%)	14 (14.1%)	1.000
Adenosquamous cancer, *N* (%)	4 (4.1%)	3 (3.1%)	1.000
**FIGO STAGE**			
IA2-IB1, *N* (%)	72 (72.7%)	73 (73.7%)	1.000
IB2-IIA2, *N* (%)	27 (27.3%)	26 (26.3%)	1.000
Tumor size	3.00 (1.30–4.00)	2.50 (1.00–4.00)	0.704
Patients with tumor size > 2 cm, *N* (%)	53 (53.5%)	50 (50.5%)	0.776

*SD, standard deviation; BMI, body mass index; SCCAg, squamous cell carcinoma antigen; CA125, serum cancer antigen 125; FIGO, International Federation of Gynecology and Obstetrics*.

Table 2 shows the postoperative pathological high-risk and intermediate-risk factor information for the two groups. Patients, with positive pelvic nodes, positive surgical margin, and/or positive parametrium, are considered to have a high-risk disease (7). Owing to the application of a propensity-matching algorithm, baseline characteristics and high risks were similar between groups. The “Sedlis Criteria” considers stromal invasion, lymphatic space involvement and primary tumor size to be intermediate risk factors (8). Regarding intermediate risk factors, there was no significant difference between the two groups. Regarding the adjuvant treatment, which includes neoadjuvant chemotherapy, adjuvant chemotherapy, and adjuvant radiation, the proportion of patients in two groups was not significantly different.

**Table 2:** Pathological high-risk and intermediate-risk factors.

	**Open abdomen** **(*N* = 99)**	**Laparoscopy** **(*n* = 99)**	***P* value**
Pelvic lymph node metastasis, *N* (%)	10 (10.1%)	11 (11.1%)	1.000
Parametrium invasion, *N* (%)	2 (2.0%)	0 (0.0%)	0.497
Vaginal cuff invasion, *N* (%)	3 (3.0%)	1 (1.0%)	0.621
Deep myometrial invasion, *N* (%)	43 (43.4%)	37 (37.4%)	0.469
Lymphovascular space invasion, *N* (%)	34 (34.3%)	23 (23.2%)	0.116
Neoadjuvant chemotherapy, *N* (%)	20 (20.2%)	20 (20.2%)	1.000
TP	17 (85.0%)	16 (80.0%)	>0.999
Others	3 (15.0%)	4 (20.0%)	>0.999
Adjuvant chemotherapy, *N* (%)	22 (22.2%)	21 (21.2%)	1.000
TP	18 (81.8%)	19 (90.5%)	0.705
Others	4 (18.2%)	2 (9.5%)	0.705
Adjuvant radiation, *N* (%)	64 (64.6%)	50 (50.5%)	0.061
Concurrent DDP-based chemotherapy	40 (62.5%)	27 (54.0%)	0.470
Others	24 (37.5%)	23 (46.0%)	0.470

*TP, paclitaxel and cisplatin; DDP, cisplatin*.

**Figure 2:**
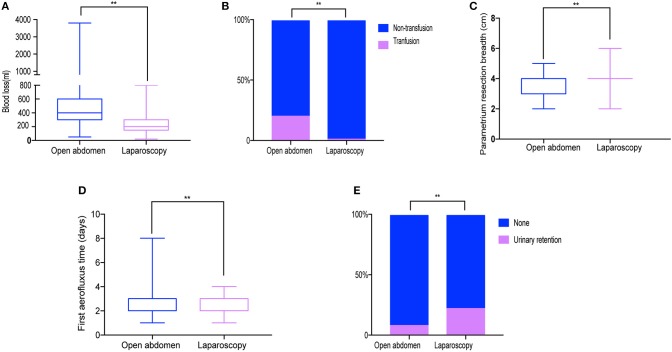
Surgery-related outcomes. **(A)** Blood loss. **(B)** Transfusion rate **(C)** Resected parametrium breadth. **(D)** Aerofluxus time. **(E)** Urinary retention rate. **P < 0.01.

Table 3 summarizes surgery-related and oncological outcomes for both groups. Regarding intraoperative outcomes, the mean volume of blood loss was significantly lower in LRH (200 mL) than ARH (400.00 ml; *P* < 0.001; Figure 2) with the transfusion rate also being lower for LRH (1.0%) than ARH (20.2%; *P* < 0.001; Figure 2). The resected parametrium in LRH was broader in LRH than ARH (*P* < 0.001; Figure 2). The median operative time, and the number of resected lymph nodes showed no significant difference between the two groups (*P* = 0.377 and 0.850, respectively). In terms of postoperative outcomes, the first aerofluxus time in the LRH (2 days) group was shorter (3 days) than that in the ARH group (*P* < 0.001; Figure 2). Time to removal of the first urinary catheter after surgery was not different between the two groups (*P* = 0.189), although the rate of urinary retention was higher in the LRH (22.2%) than ARH (8.1%) group (*P* = 0.009; Figure 2).

**Table 3:** Surgery-related and oncological outcomes of the patients.

	**Open abdomen** **(*N* = 99)**	**Laparoscopy** **(*n* = 99)**	***P* value**
Operating time (min)	165 (150.00–180.00)	180 (160.00–200.00)	0.377
The number of resected lymph nodes	27 (20.00–34.00)	26 (22.00–34.75)	0.850
Blood loss (ml)	400.00 (300.00–600.00)	200.00 (150.00–300.00)	<0.001
Transfusion, *N* (%)	20 (20.2%)	1 (1.0%)	<0.001
Severe complications in 30 postoperative days, *N* (%)	19 (19.2%)	22 (22.2%)	0.726
Severe complications after 30 postoperative days, *N* (%)	2 (2.0%)	5 (5.1%)	0.445
Unexpected second surgery, *N* (%)	3 (3.0%)	4 (4.0%)	1.000
Unexpected second hospitalization, *N* (%)	2 (2.0%)	5 (5.1%)	0.445
Postoperative first aerofluxus time (days)	3.00 (2.00–3.00)	2.00 (2.00–3.00)	<0.001
Length of hospital stay(days)	11.00 (9.00–17.00)	10.00 (8.00–16.00)	0.224
Postoperative first uterine catheter removal time (days)	14.00 (14.00–14.00)	14.00 (11.50–14.00)	0.189
Urine retention, *N* (%)	8 (8.1%)	22 (22.2%)	0.009
Relapse, *N* (%)	4 (4.0%)	8 (8.1%)	0.375
**RELAPSE SITE**			
Pelvic cavity	2 (50.0%)	5 (62.5%)	>0.999
Distance	2 (50.0%)	3 (37.5%)	>0.999
Mortality, *N* (%)	3 (3.0%)	4 (4.0%)	1.000
Follow-up time (months)	69.00 (61.00–76.00)	59.00 (53.00–67.00)	

There was no difference between the two groups in terms of length of hospital stay, rate of unexpected second hospitalization and rate of unexpected second surgery rate (*P* = 0.224, 0.445, and 0.375, respectively). The detailed information of surgery-associated complications (grade ≥ 3) is presented in Table 4. Overall, 19 adverse events were associated with LRH and 22 with ARH, over a period of 30 days after surgery, which was not significantly different between the two groups (*P* = 0.726). In the time period after 30 days, five adverse events were noted in the LRH group and two in the ARH group, again this difference not being significant (*P* = 0.445).

**Table 4:** The detail information of surgery-related complications in two groups.

	**Open abdomen** **(*n* = 99)**	**Laparoscopy** **(*n* = 99)**
Severe complications in 30 postoperative days, *N* (%)	19 (19.2%)	22 (22.2%)
Vesicovaginal fistula	0	2
Ureteral fistula	1	1
Intestinal obstruction	5	0
Poor wound healing	6	1
Pelvic lymphocyst (Puncture needed)	1	1
Fever/Infection	6	15
Deep vein thrombosis	0	1
Rectovaginal fistula	0	1
Severe complications after 30 postoperative days, *N* (%)	2 (2.0%)	5 (5.1%)
Hydronephrosis (Double J needed)	2	4
Urinary incontinence	0	1

**Figure 3:**
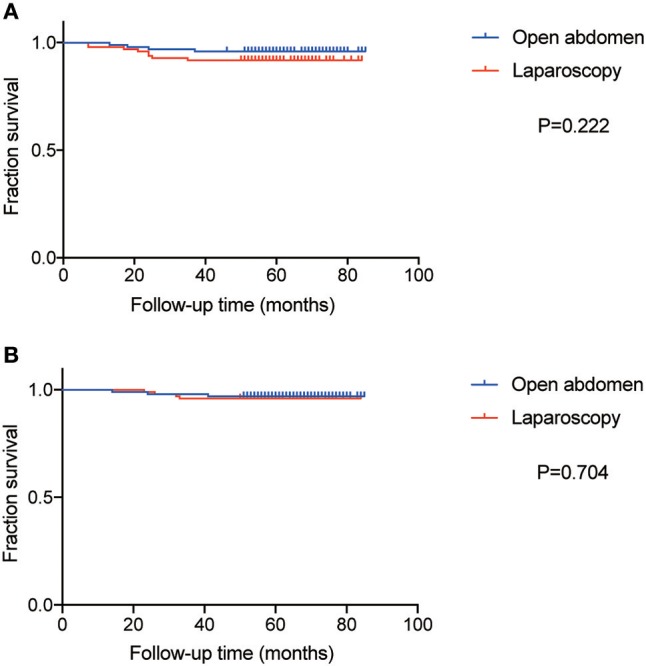
The curve of Disease-free survival (DFS) and overall survival (OS). **(A)** The curve of DFS. **(B)** The curve of OS.

The median follow-up time of patients was 69 months in the ARH group and 59 in the LRH group. The DFS and OS curves, shown in Figure 3, were not significantly different between the two groups (*P* = 0.222 and 0.704, respectively). The rates of DFS at 3-years in ARH and LRH were 96.0 and 92.0%, respectively. And the rates of OS at 3-year in ARH and LRH were 97.0 and 96.0%, respectively. Moreover, by IPWT, laparoscopy was also not associated with poorer survival in terms of DFS (HR 1.52, CI 0.799–2.891, *P* = 0.202) or OS (HR 0.942, HR 0.425–2.09, *P* = 0.883).

## A Review

A literature search was performed in PubMed, Embase and Web of Science, using the following search words: “uterine cervical neoplasm,” “laparotomy,” “laparoscopy,” and “minimally invasive surgical procedures.” The search was limited to publications between January 2014 and May 2019. Studies included in our review had to meet all of the following criteria: (a) uterine cervical cancer; (b) radical hysterectomy and lymphadenectomy; (c) data including the comparison of laparotomy and laparoscopy; (d) date including oncologic survival outcomes; (e) studies published in English. In total 13 articles were included (1, 9–20) with details shown in Supplementary Table 1.

## Discussion

Our findings provide evidence of comparable or better surgical and oncologic outcomes for LRH than ARH, with the exception of a higher rate of urinary retention after LRH. We note the details of our study design that strengthen our evidence. First, acknowledging that these outcomes will be influenced by surgeons' experience, particularly between institutions (21–23). We conducted a single site study, with all procedures performed by experienced oncologic gynecological surgeons. Also, considering the learning curve required for laparoscopic procedures (21), we selected patients who underwent LRH or ARH after January 2012, when laparoscopic technique was well-established in our center, thus excluding effects of inexperience on LRH outcome. Lastly, we also used propensity score matching (24) and IPWT to balance the two comparison groups on all major factors known to influence outcomes. To the best of our knowledge, this is the largest single site study, with propensity score matching and IPWT, to have compared LRH to ARH in terms of surgical and oncologic outcomes.

Our findings of better intra-operative outcomes for LRH than ARH are consistent with previous studies (10, 13, 25), including a smaller volume of blood loss and a lower transfusion rate. These findings reflect the better ability to identify small vessels under the magnification provided by an optical system on the laparoscope, as well as the use of the argon-beam coagulator during surgery (26). Our finding of a shorter time to first flatus in the LRH than ARH group, indicative of a relatively shorter post-operative recovery, is also consistent with previous studies (27, 28).

Gynecological surgery for malignancy is associated with a high incidence rate of pelvic disorder (29). Urinary retention is one of symptoms related to urinary dysfunction associated with pelvic floor disorder, which notably occurs with cervical cancer surgery (30). Urinary retention leads to delay in ultimate removal of the urinary catheter, which increases the risk of urinary tract infection and negatively impacts the post-operative quality of life (30). It is important to note, however, that the definition of urinary retention varies across studies. Using the definition of a post-void residual urine volume >100 ml, Ceccaroni et al. reported a prevalence rate of urinary retention of 39%, 28 days after laparoscopic type III hysterectomy (31). Using a similar definition, the rate of urinary retention in LRH was 22.2%. Moreover, the rate in LRH was significantly higher than the 8.1% rate in the ARH group. We note that our finding was not consistent with those of previous studies (27, 32). One previous study reported a significantly longer duration of catheterization than after open laparoscopic surgery (27). A longer duration of catheterization after open surgery was similarly reported in another study, where urinary retention was defined by an absence of voiding > 6 h after Foley catheter removal, with a concomitant estimation of bladder filling >300 ml on ultrasound examination (32). Differences in definition of urinary retention and the broader resection parameter LRH may be the reason why our rate of urinary retention was higher for LRH than ARH.

Differences in oncological outcomes between LRH and ARH are essential to consider and opinions vary from different studies (1, 9–20). After the consternation brought to light by the study of Ramirez et al. (1), a prospective randomized international phase III trial, gynecologists began to think how do we proceed in the face of these data (33). More than 85% of cervical cancer cases occur in developing countries (34). China as a developing country has multiple cases of patients with cervical cancer. How do gynecologists in China proceed in the face of these data?

First, we analyzed the results in our institution in our study, the rates of DFS at 3-years in ARH and LRH were 96.0 and 92.0%, respectively and the rates of OS at 3-years in ARH and LRH were 97.0 and 96.0%, respectively, which were comparable to that seen in the open abdomen group in the studies of Ramirez et al. (1) and those in the study of Martin-Hirsch et al. (33). No significant difference was found between the ARH group and LRH group in our study.

**Figure 4:**
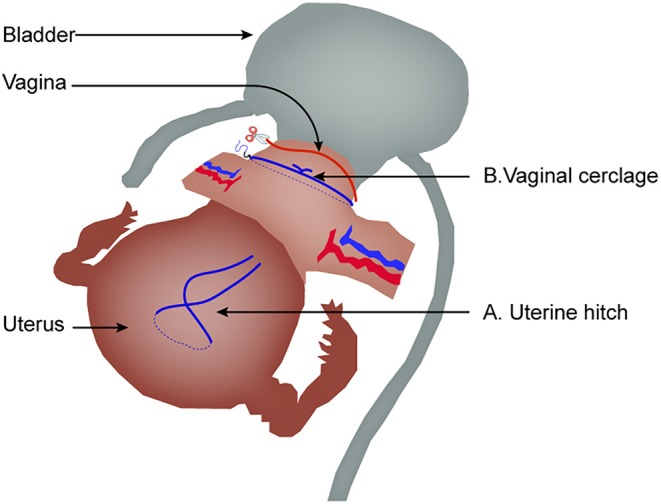
The evolved expertise in laparoscopic radical hysterectomy. **(A)** Uterine hitch technique replacing uterine manipulator. **(B)** Vaginal cerclage before creating an incision in the vaginal wall.

Second, as shown in Figure 4, technical expertise in China has evolved, moving away from using a uterine manipulator to a hitch technique (35). Moreover, before creating an incision in the vaginal wall, vaginal cerclage is now performed to avoid tumor exposure to the abdominal cavity, adhering to the non-tumor principle. Whether the evolving expertise could be beneficial to oncological outcome or not remains to be investigated.

In conclusion, in our study, LRH was associated with less intra-operative complications and better postoperative recovery than ARH, with no significant difference in oncologic outcomes. As technological expertise with LRH continues to grow, future studies are needed to continue to monitor and evaluate outcomes.

## Data Availability Statement

All datasets generated for this study are included in the article/Supplementary Material.

## Ethics Statement

This study was approved by the Peking Union Medical College Hospital Ethics Review Board. Preoperatively, all patients provided written informed consent for data collection for research purposes.

## Author's Note

Compared with abdominal radical hysterectomy, laparoscopic radical hysterectomy provided better intra-operative and post-operative outcomes, with no significant difference in oncologic outcomes and survival.

## Author Contributions

All authors listed have made a substantial, direct and intellectual contribution to the work, and approved it for publication.

### Conflict of Interest

The authors declare that the research was conducted in the absence of any commercial or financial relationships that could be construed as a potential conflict of interest.
