# Supplementary material for: Laparoscopic vs. Open Abdominal Radical Hysterectomy for Cervical Cancer: A Single-Institution, Propensity Score Matching Study in China
Source: Front Oncol. 2019 Oct 30;9:1107. doi: 10.3389/fonc.2019.01107 (PMC6833183; doi:10.3389/fonc.2019.01107)
Supplement: Supplementary file 1 [file Data_Sheet_1.docx]

| **First author** | **Publication Year** | **Country** | **Institution** | **Study design** | **Years of Study** | **FIGO Stage (2009)** | **No. of Pats with ARH** | **No. of Pats with LRH** | **Favor** | **Outcomes** | **Follow up (months)** |
| --- | --- | --- | --- | --- | --- | --- | --- | --- | --- | --- | --- |
| Ratiu, D (9) | 2019 | Germany | Single | Retrospective study | From 2011 to 2017 | Stage IA1 to IIB | 41 | 34 | LRH | 1. OS rate: 94.1% in LRH; 85.4% in ARH (p=0.311). 2. DFS rate: 94.1% in LRH; 75.6% in ARH (p=0.049) | Mean, 41 |
| Gil-Moreno, A (10) | 2019 | Spain | Single | Prospective controlled study | From 1999 to 2016 | Stage IA2 to IIB | 76 | 90 | Equal | 1. Recurrence: 13/90(14.4%) in LRH; 11/76 (14.5%) in ARH 2. Death: NA | Median,112.4 |
| Ramirez, P (1) | 2018 | International | Mutiple | Prospective randomised study | From 2008 to 2017 | Stage IA1 to IB1 | 319 | 312, MIS; 84.4% LRH | ARH | 1. DFS rate at 4.5y: 86.0% in MIS; 96.5% in ARH, −10.6% (95%CI, −16.4 to −4.7) 2. DFS rate at 3y: 91.2% in MIS; 97.1% in ARH; HR, 3.74 (95% CI, 1.63 to 8.58); 3. OS rate at 3y: 93.8% in MIS; 99.0% in ARH; HR, 6.00 (95% CI, 1.77 to 20.30). | Median, 30 |
| Melamed, A (11) | 2018 | United States | Mutiple | 1.Cohort study, using inverse probability of treatment propensity-score weighting 2. Time-series analysis using SEER | 1. From 2010 to 2013 2. From 2000 to 2010 | Stage IA2 or IB1 | 1236 | 1225, MIS | ARH | 1. Death rate at 4y: 9.1% in MIS; 5.3% in ARH 2. The adoption of MIS was associated coincided with the beginning of a decline in the 4-year relative survival rate of 0.8% (95% CI, 0.3 to 1.4) per year | Meidan, 45 |
| Corrado, G (12) | 2018 | Rome | Double | Retrospective study | From 2001 to 2016 | Stage IB1 | 101 | 152, LRH; 88, RRH | Equal | 1. DFS rate at 5y: 91.3% in ARH; 87.2% in LRH; 89.5% in RRH 2. OS rate at 5y: 88.7% in ARH; 89.7% in LRH; 88.8% in RRH 3. The Kaplan-Meier curves: RFS between ARH and MIS (P = 0.03), OS between the 3 groups (P = 0.69) | Median, 82.1 in ARH; Median, 41.7 in LRH; Median, 46.6 in RRH |
| Diver, E (13) | 2017 | United States | Double | Retrospective cohort study | From 2000 to 2013 | Stage IA to IIB | 282 | 30, LRH; 71, RRH | Equal | 1. Recurrence rate: 5.0%, in MIS; 6.4% in ARH (p = 0.86) 2. OS rate: p = 0.29 | Mean, 61.2 |
| Laterza, R (14) | 2016 | Italy | Single | Retrospective study | From 1997 to 2014 | Stage IA1, IA2, IB1 and IIA1 | 68 | 82 | Equal | 1. Recurrence rate: 9/68 in ARH; 13/82 in LRH, P = 0.17 2. Time interval to recurrence and recurrence site: no difference | Median, 121.2 in ARH; Meidian, 43.5 in LRH. |
| Corrado, G (15) | 2016 | Rome, Italy. | Single | 1. Prospective nonrandomized analysis of all cases of RRH 2. To compare collected data with 2 historical cohort of women who underwent LRH or ARH at the same institution | 1.RRH: From 2010 to 2013 2.LRH: From 2004 to 2012 3.ARH: From 2001 to 2011 | Stage IB2-IIB | 43 | 41, LRH; 41, RRH; | Equal | 1. Recurrence:8 in ARH; 4 in LRH; 5 in RRH 2. Death: 7 in ARH; 4 in LRH; No in RRH 3. DFS rate at 3y: 83.7% in ARH; 89.2% in LRH; 86.7% in RRH 4. OS rate at 3y: 90.5% in ARH; 91.9% in LRH; 100% in RRH | Median,101.4 in ARH; Median, 60.1 in LRH; Median, 30 in RRH. |
| Arispe, C (16) | 2016 | Spain | Single | Retrospective study | From 1990 to 2013 | Stgae IA1-IIA1 | 55 | 47 | ARH | 1. DFS: significant better outcomes in ARH compared to LRH (P=0.015) 2. No data about mortality. | Mean, 109.9 |
| Xiao, M (17) | 2015 | China | Single | Retrospected study | From 2001 to 2014 | Stage IA–IIB | 48 | 106 | Equal | 1. DFS rate: 88.9% in ARH; 89.7% in LRH (P= 0.39) 2. OS rate: 91.3% in ARH, 90.2% in LRH (P = 0.40) | Mean, 64.6 in ARH; Mean, 48.2 in LRH |
| Bogani, G (18) | 2015 | Italy | Single | Retrospected study | T1 (2000-2003), ARH was the standard of care; T2 (2004-2007), a systematic implementation of LRH; T3 (2008-2011), LRH was the standard of care | Not in detail | Not in detail | Not in detail | Equal | DFS rate a 5y: no difference Time interval to recurrence and Recurrence site: no difference | Mean, 105.9, 79.2, and 40.9, respectively. |
| Toptas, T (19) | 2014 | Turkey | Single | Retrospective study | From 2007 to 2010 | Stage IA2-IB1 | 46 | 22 | Equal | DFS rate at 3y: 90.6% in ARH; 86.1% in LRH(p=0.32) OS rate at 3y: 95.4% in ARH; s 100% in LRH(P=0.82) | Median, 43.50 in ARH; Median, 42.50 in LRH; |
| Chen, C (20) | 2014 | Taiwan | Single | Retrospective study | NA | Stage IA to IIB | 44 | 32, LRH; 24, RRH. | Equal | DFS rate: 90.9% in ARH; 90.6% in LRH; 95.8% in RRH; no significant difference. | Mean, 37.1 in ARH; Mean, 34.6 in LRH; Mean, 13.9 in RRH. |

**Supplementary Table 1. Characteristics of the previously reported studies. FIGO, International Federation of Gynecology and Obstetrics; No, number; Pats, patients; ARH, open abdomen radical hysterectomy; LRH, laparoscopic radical hysterectomy; RRH, Robotic radial hysterectomy; MIS, minimally invasive surgery; OS, overall survival; DFS, Disease-free survival; y, years;**
